# Longitudinal associations between intergroup contact and intergroup trust among adolescents in ethnic regions of China

**DOI:** 10.1038/s41598-024-66646-1

**Published:** 2024-07-10

**Authors:** Xiaojiao Yuan, Tianyang Lei, Ying Su, Xiaoxia Li, Lan Zhu

**Affiliations:** 1 School of Education and Psychology, Southwest Minzu University, Xihanggang Street, Chengdu, 610225 China; 2 Key Research Institute of Humanities and Social Sciences of State Ethnic Affairs Commission, Southwest Minzu University, Chengdu, China

**Keywords:** Intergroup contact, Intergroup trust, Loneliness, Adolescents in ethnic regions, Psychology, Human behaviour

## Abstract

Intergroup trust is a crucial psychological foundation for promoting ethnic unity and maintaining social stability. This study explored the dynamic relationship between intergroup contact and trust among adolescents in ethnic regions of China and sought to uncover the mechanisms behind this relationship. Through a two-wave longitudinal survey of 679 adolescents, employing the Intergroup Contact Experience Scale, the Intergroup Trust Scale, and the UCLA Loneliness Scale, the study yielded several findings: (1) Over the year, a significant increase in intergroup contact was observed among the adolescents. Notably, junior high students demonstrated a marked rise in intergroup trust, whereas no significant change was discernible in high school students. (2) Cross-lagged analysis indicated that early intergroup contact significantly predicted subsequent intergroup trust and loneliness. Initial loneliness also forecasted future intergroup trust, yet early intergroup trust did not predict later intergroup contact or loneliness. (3) Loneliness served as a partial mediator in the longitudinal link between intergroup contact and trust among these adolescents. These findings reinforce the premise that in China’s ethnic regions, intergroup contact is a precursor to intergroup trust, both directly enhancing trust among adolescents and indirectly by reducing loneliness.

## Introduction

Intergroup trust is a psychological characteristic and relational aspect that reflects mutual understanding, respect, and trust among different ethnic groups during intergroup interactions^1^. In China, enhancing intergroup trust is considered a crucial task in the current governance of national ethnic affairs^2^ and an effective strategy for promoting stability and unity in ethnic regions^3^. Despite this, much of the empirical research on intergroup trust has been conducted in Western countries, particularly within immigrant populations. Although research within the Chinese context has started to increase, these studies have generally focused on university students^4,5^ and have been predominantly cross-sectional^6^.

Adolescence is a critical period for peer interactions and identity formation, as well as a pivotal time for the development of intergroup attitudes. Research indicates that adolescents have a greater potential for change in intergroup attitudes compared to adults^7^, with the effect size being approximately twice as large in adult samples^8^. Moreover, the development trends of intergroup attitudes among adolescents vary across different socio-cultural backgrounds. For instance, a study in Turkey revealed an increase over time in tendencies to avoid intergroup contact and negative intergroup attitudes among adolescents^9^, while other research has shown positive changes over time in the intergroup attitudes between native and immigrant adolescents in Italy and the UK^10,11^. Therefore, exploring the developmental patterns of intergroup trust among adolescents in ethnic regions within China’s unique socio-cultural context is of significant importance for enriching the theoretical contributions of this field and for enhancing the practice of promoting ethnic unity.

### Socio-cultural and interethnic interaction background of ethnic regions in China

China is a unified multi-ethnic nation, with ethnic autonomous areas constituting 64% of the country’s territory. These ethnic regions are distinct in many aspects such as cultural traditions and ethnic interactions^12^. From a broad socio-cultural perspective, the 56 ethnic groups in China have historically built a relationship of mutual reliance, joint progress, and shared development. This relationship has evolved into a pattern of unity in diversity within the Chinese nation^13^, fostering a strong collective identity—the Chinese national identity. In terms of the intergroup interaction environment, unlike Western contexts where transnational immigrants enter new mainstream cultural societies and interact with dominant ethnic groups, in China, ethnic regions predominantly comprise minority ethnic group settlements operating under a system of regional ethnic autonomy^14^. In Sichuan Province, the Aba Tibetan and Qiang Autonomous Prefecture and the Liangshan Yi Autonomous Prefecture serve as examples of the complexity of China’s ethnic composition. While the Aba Prefecture is primarily inhabited by the Tibetan and Qiang ethnic groups, it also hosts a smaller population of Han and other ethnic minorities. Likewise, the Liangshan Prefecture is mainly Yi but includes a smaller representation of Han and other ethnic minorities. Such demographic configurations lead to intricate patterns of interethnic interactions. These dynamics foster an environment of intergroup contact for adolescents in China’s ethnic regions that is notably different from the patterns of interaction often studied in Western contexts of immigration.

### The relationship between intergroup contact and intergroup trust

Intergroup contact is recognized as one of the most significant factors influencing intergroup attitudes. The Intergroup Contact Hypothesis postulates that intergroup hostility can be reduced and positive out-group attitudes formed when different groups engage in intergroup contact under optimal conditions^15^. Decades of research have provided support for this theory. A meta-analysis by Pettigrew and Tropp^8^ of 515 studies across 38 countries found a significant negative correlation between intergroup contact and prejudice. The quantity of contact, such as the frequency of interactions with members of outgroups, and the quality of contact, such as the positivity and cooperativeness of intergroup interactions, are both associated with more favorable attitudes towards out-groups^6,16^. Studies on Chinese university student populations have also shown that intergroup contact can reduce negative stereotypes held between different groups^17^ and foster positive trust attitudes^18^. However, some researchers argue that a selection bias effect is plausible, where intergroup attitudes could influence intergroup contact^19,20^. When individuals have more trust in an out-group and positive expectations about intergroup contact, they may be more inclined to seek out such interactions.

Existing research has tested the causal relationship from intergroup contact to trust through experimental paradigms and found that intergroup contact can influence individuals’ attitudes towards out-groups^21,22^. However, the ecological validity of such artificially manipulated and controlled experimental designs is limited, making it difficult to reveal the true effects of intergroup contact in natural settings. Moreover, due to the difficulty in manipulating intergroup attitudes, the reverse relationship from intergroup trust to contact has not been sufficiently explored.

Longitudinal studies are particularly effective in examining the process of intergroup contact in natural settings because they can provide temporal information to assess whether changes in intergroup contact precede changes in intergroup attitudes. The cross-lagged panel model (CLPM) is an effective method for exploring dynamic relationships between variables. By constructing paths from the baseline level of a variable to its subsequent level (i.e., autoregressive effects), as well as paths from the baseline level of one variable to the subsequent level of another variable (i.e., cross-lagged effects), the CLPM can reveal diachronic effects between variables, demonstrate the bidirectional dynamic relationship over time, and provide a basis for validating causal relationships^23,24^. However, longitudinal designs and cross-lagged studies in the field of intergroup attitudes are still relatively scarce and have yielded divergent conclusions. For instance, Binder et al. conducted a two-wave longitudinal study surveying groups of secondary school students in Germany, Belgium, and the United Kingdom, which included 512 ethnic minority members and 1143 ethnic majority members. The study found a bidirectional relationship between intergroup contact and prejudice, indicating that intergroup contact can reduce prejudice, and conversely, prejudice can also reduce intergroup contact^19^. In another study, Dhont et al. employed a two-wave design to examine intergroup contact using self-reports and observer reports among a sample of 65 Belgian young adults and their 172 close friends. The results indicated that intergroup contact predicted subsequent reductions in intergroup prejudice, but intergroup prejudice did not effectively predict subsequent intergroup contact^25^. However, within the socio-cultural context of China, the dynamic relationships between intergroup contact and intergroup attitudes have been minimally explored. This study seeks to bridge this gap using a two-wave longitudinal survey and the CLPM.

### The relationship between intergroup contact, intergroup trust, and loneliness

Researchers have explained from various angles how intergroup contact influences intergroup trust. For instance, intergroup contact can help to reduce prejudice and negative stereotypes^26^, produce a decategorization effect^27,28^, and lessen interaction anxiety^29^, among other benefits. Building on the Intergroup Contact Hypothesis^15,30^, Social Identity Theory^31^, and considering the socio-cultural background of interethnic interactions among adolescents in China’s ethnic regions, we postulate that the effect of intergroup contact on intergroup trust may also be related to the emotional dimension of loneliness. Based on the pattern of the Chinese nation’s unity in diversity and relevant ethnic policies, intergroup contact among adolescents in China’s ethnic regions largely meets the optimal contact conditions^6^. Positive intergroup contact can alleviate loneliness by providing opportunities for social interaction and support^32,33^. Through positive intergroup contact, adolescents can gradually establish cross-ethnic friendships^34,35^, which can help to reduce feelings of loneliness. Furthermore, in the context of the new era of forging a strong sense of community among the Chinese nation, government, community, schools, media, and other entities vigorously promote and advance interethnic interactions^36^. Intergroup contact can also expand the perceived interethnic boundaries for adolescents in ethnic regions, helping them to gain more support and a sense of belonging to a common identity^37^, which similarly contributes to reducing adolescent loneliness. This reduction in loneliness, in turn, enhances their trust towards out-group members, as positive social experiences can alleviate fears and anxieties associated with intergroup interactions^29^, while simultaneously fostering positive feelings towards out-groups^34,35^.

### The present study

The present study employs a longitudinal design to investigate the developmental characteristics of intergroup contact and intergroup trust among adolescents in China’s ethnic regions, to analyze the dynamic interplay between the two, and to test the potential mediating role of loneliness in their longitudinal association. Drawing on prior research, this study proposes the following hypotheses: H1: Intergroup contact among adolescents in China’s ethnic regions will increase over time, and intergroup trust will be gradually strengthened; H2: Intergroup contact serves as an antecedent variable to intergroup trust within a cross-lagged framework; H3: Loneliness mediates the longitudinal relationship between intergroup contact and trust among adolescents in China’s ethnic regions.

## Methods

### Participants

Based on the ethnic regional characteristics and the distribution of minority populations in Sichuan Province, China, this study conducted surveys in one ethnic school each within Liangshan Yi Autonomous Prefecture and Aba Tibetan and Qiang Autonomous Prefecture. Employing cluster sampling, the study initially enrolled 777 adolescents from 17 classes, with data collected at two points over the course of one year. Class adjustments and various absenteeism led to the loss of 98 participants by the time of follow-up. Independent sample t-tests were conducted on the baseline data for both the retained and lost participant groups. The results indicated no significant differences between the retained and lost samples of each school in terms of intergroup contact, loneliness and intergroup trust scores at Time 1(T1), suggesting that there was no selective attrition of participants. The effective sample consisted of 679 adolescents who participated in both measurements. The breakdown of the sample was as follows: 297 males (43.7%), and 382 females (56.3%); 313 junior high students (46.1%), and 366 senior high students (53.9%); the ethnic composition included Yi (329 individuals, 48.5%), Qiang (168 individuals, 24.7%), Tibetan (127 individuals, 18.7%), Han and other minorities (55 individuals, 8.1%). The average age at T1 was 15.68 ± 1.89 years. Participants had a good command of Mandarin Chinese, which was the language of instruction in the schools, thus the study was conducted using Mandarin Chinese versions of the questionnaires.

### Instruments

*Intergroup Contact* The intergroup contact was assessed using the Intergroup Contact Experience Scale revised by Yang et al.^38^, which was originally developed by Islam and Hewstone^39^. The scale consists of 9 items scored on a 7-point scale and includes two subscales: quantity of contact and quality of contact. Contact quantity refers to the frequency and number of interactions with classmates, roommates, and friends from other ethnic groups; contact quality refers to the equality, cooperativeness, intimacy, and pleasure experienced during interactions with the out-group. An example item for quantity of contact is: “How often do I converse with students from other ethnic groups?” For quality of contact, an example item is: “My relationship with friends from other ethnicities is enjoyable/unenjoyable.” The higher the overall average score, the greater the degree of intergroup contact. In this study, the Cronbach’s alpha coefficient for intergroup contact was 0.91 at baseline and 0.92 at follow-up (Supplementary Information file).

*Intergroup Trust* Intergroup trust was measured using the Intergroup Trust Scale revised by Liu^40^, originally developed by Voci and Hewstone^41^. Participants were asked to assess their attitudes towards other ethnicities in four areas: “trust,” “reliability,” “lack of trust,” and “suspicion,” rating them on a scale from 1 “never feel this way” to 7 “always feel this way.” After reversing scores for reverse-scored items, calculate the average. A higher score indicates stronger trust towards out-groups. In this study, the Cronbach’s alpha coefficient for intergroup trust was 0.78 at baseline and 0.77 at follow-up.

*Loneliness* Loneliness was assessed using the UCLA Loneliness Scale developed by Russell et al.^42^. The scale includes 20 items, scored on a 4-point scale from 1 “Never” to 4 “Always”. An example item is: “How often do you feel that your relationships with those around you are harmonious?” Higher scores indicate higher levels of loneliness. In this study, the Cronbach’s alpha coefficient for loneliness was 0.89 at baseline and 0.91 at follow-up.

### Procedure

This study was reviewed by the ethics committee of the researchers’ affiliated institution and proceeded with informed consent obtained from the administrative leaders of the schools, the students, and their parents. By selecting a revised research tool under Chinese cultural conditions, conducting preliminary baseline answering, and formulating survey instructions and precautions, we have endeavored to ensure the cultural adaptability of the research tool. The initial round of data collection was conducted from May to June 2022, with pencil-and-paper tests administered in a classroom setting by class. One year later, a follow-up test was carried out in the same sampled classes. Both data collection processes were jointly organized by graduate students in psychology and school teachers, who supervised and guided the questionnaire completion on-site. All questionnaires were collected on the spot after inspection. Each participant received a gift after completing the questionnaire each time.

### Data processing and statistical analysis

Firstly, descriptive statistical analysis and multivariate repeated measures ANOVA were used to reveal changes in intergroup contact and trust among ethnic minority adolescents over a one-year interval. Subsequently, correlational analyses of the study variables were conducted, and the CLPM was used to examine the dynamic relationships between intergroup contact, intergroup trust, and loneliness. On this basis, the mediating effect of loneliness in the longitudinal relationship between intergroup contact and trust was tested using structural equation modeling.

Since the data collected in this study were all from individual self-reports, Harman’s single-factor test^43^ was used to test for common method bias. The results indicated that there were 13 factors with eigenvalues greater than 1, and the percentage of total variance explained by the first factor was 24.92%, which is below the critical threshold of 40%. Therefore, there was no serious common method bias present in the study.

### Ethics approval

This study was strictly followed the Declaration of Helsinki and approved by the Professor and Ethics Committee, School of Education and Psychology, Southwest Minzu University (Approval Number: JX2022016). In this study, we obtained informed consent from the administrative leaders of the schools, the students, and their parents.

## Results

### Development of intergroup contact and trust among adolescents in ethnic minority areas

To examine the evolution of intergroup contact and trust among adolescents in ethnic minority regions, a 2 (time of testing) × 2 (educational stage) multivariate repeated measures ANOVA was executed. The results indicated a significant main effect of time [*F*(2,667) = 15.84, *η*^*2*^ = 0.05, *p* < 0.001]. After one year, intergroup contact among ethnic minority adolescents had significantly increased [*F*(1,668) = 31.64, *η*^*2*^ = 0.05, *p* < 0.001], but there was no overall significant change in intergroup trust [*F*(1,668) = 0.91, *η*^*2*^ = 0.001, *p* > 0.05]. However, there was a significant interaction between measurement time and educational stage on intergroup trust [*F*(1,668) = 8.67, *η*^*2*^ = 0.01, *p* < 0.01], with a significant increase in trust among junior high school students [*t*(307) =  − 2.59, *d* =  − 0.15, *p* < 0.05], while there was no significant change among high school students [*t*(362) = 1.48, *d* = 0.08, *p* > 0.05]. The main effect of educational stage was significant [*F*(2,667) = 3.59, *η*^*2*^ = 0.01, *p* < 0.05], with high school students showing higher levels of intergroup contact [*F*(1,668) = 6.36, *η*^*2*^ = 0.01, *p* < 0.05] and intergroup trust [*F*(1,668) = 4.47, *η*^*2*^ = 0.01, *p* < 0.05] compared to junior high school students. Detailed developmental results of intergroup contact and trust among adolescents in different educational stages in ethnic minority areas of China are presented in Table 1.

**Table 1:** The development of intergroup contact and intergroup trust among adolescents in China’s ethnic regions.

	T1				T2			
	Junior high school students		Senior high school students		Junior high school students		Senior high school students	
	*M*	*SD*	*M*	*SD*	*M*	*SD*	*M*	*SD*
Intergroup contact	3.97	1.48	4.26	1.55	4.22	1.59	4.50	1.67
Intergroup trust	4.86	1.26	5.17	1.09	5.05	1.17	5.08	1.23

### Correlation among study variables

Pearson’s correlation analyses were conducted on the T1 and T2 measures of intergroup contact, intergroup trust, and loneliness among adolescents in ethnic minority areas, with results presented in Table 2.

**Table 2:** Correlations among study variables.

	1	2	3	4	5	6	*M*	*SD*
1 T1 intergroup contact	–						4.11	1.53
2 T1 intergroup trust	0.41***	–					5.03	1.18
3 T1 loneliness	− 0.26***	− 0.42***	–				2.15	0.50
4 T2 intergroup contact	0.76***	0.33***	− 0.25***	–			4.35	1.64
5 T2 intergroup trust	0.44***	0.44***	0.42***	0.52***	–		5.06	1.20
6 T2 loneliness	− 0.27***	− 0.30***	0.67***	− 0.33***	− 0.56***	–	2.15	0.50

***p < 0.001.

The analyses revealed significant correlations among the study variables. There were significant positive correlations between the T1 and T2 levels for each variable, as well as between T1 and T2 intergroup contact and trust. Additionally, there were significant negative correlations between T1 and T2 loneliness and both intergroup contact and trust. The correlation analysis results suggest the appropriateness of further cross-lagged analysis among these variables.

### Cross-lagged analysis of intergroup contact, intergroup trust, and loneliness

**Figure 1:**
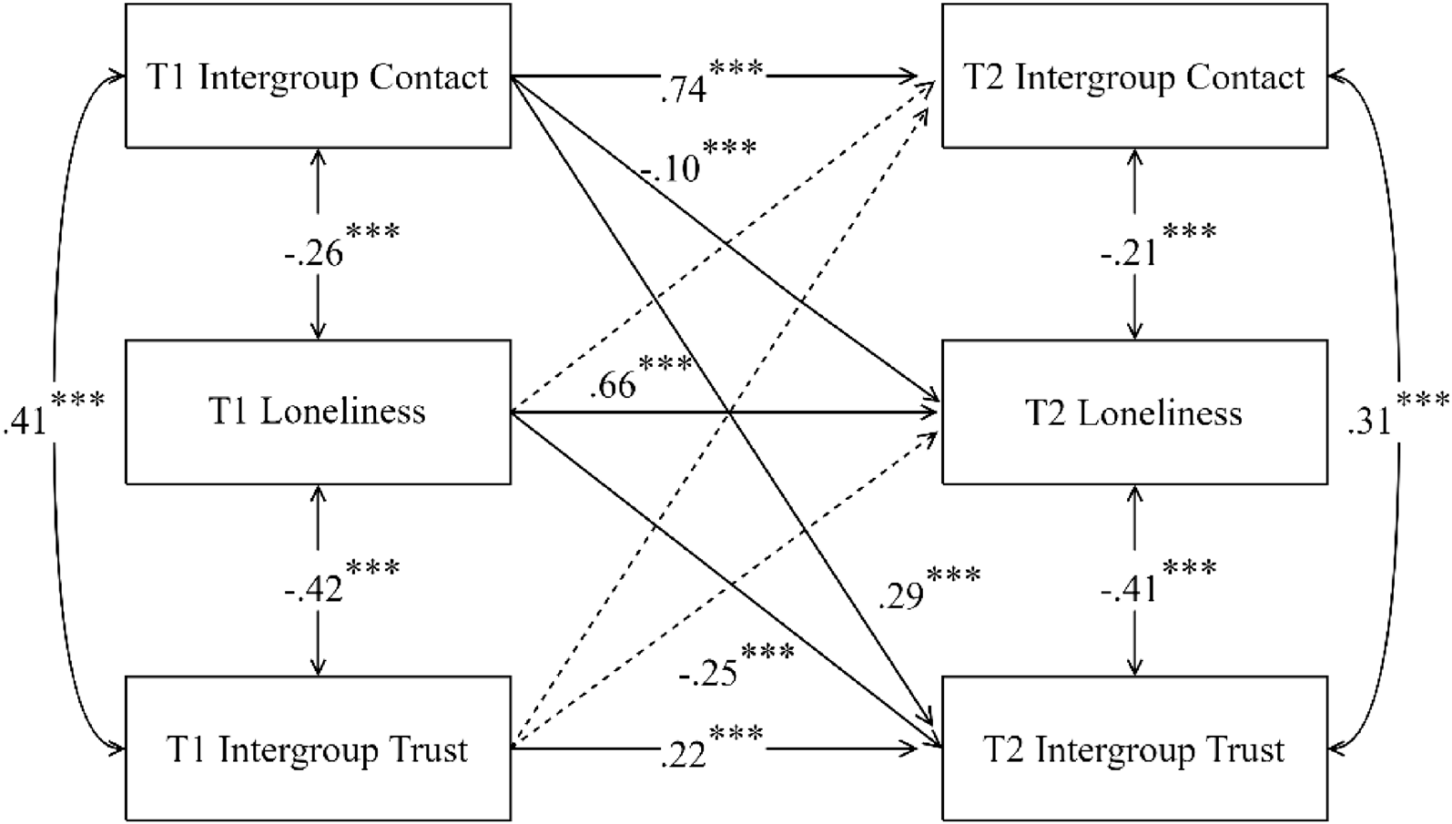
Cross-lagged analysis of study variables. Dashed lines in the model represent path coefficients that do not achieve statistical significance at the *p* < .05 level.

A cross-lagged analysis was conducted to examine the dynamic relationships between intergroup contact, intergroup trust, and loneliness among adolescents in ethnic minority areas, with results depicted in Fig. 1.

In terms of autoregressive effects, T1 levels of intergroup contact, loneliness, and intergroup trust significantly positively predicted their respective T2 levels, indicating that these constructs exhibit a certain degree of stability among ethnic minority adolescents over the course of a year. Intergroup contact and loneliness demonstrated greater stability, whereas intergroup trust was relatively less stable. In terms of cross-lagged effects, T1 intergroup contact significantly negatively predicted T2 loneliness (*β* =  − 0.10, *p* < 0.001) and significantly positively predicted T2 intergroup trust (*β* = 0.29, *p* < 0.001). However, neither T1 loneliness nor T1 intergroup trust significantly predicted T2 intergroup contact, suggesting that intergroup contact is a precursor variable in the development of loneliness and intergroup trust. High levels of intergroup contact can reduce subsequent loneliness and enhance intergroup trust. Moreover, T1 loneliness significantly negatively predicted T2 intergroup trust (*β* =  − 0.25, *p* < 0.001), but T1 intergroup trust did not significantly predict T2 loneliness, indicating that loneliness is a precursor variable in the development of intergroup trust. Lower levels of loneliness are beneficial in enhancing intergroup trust.

### Longitudinal mediation effect analysis

**Figure 2:**
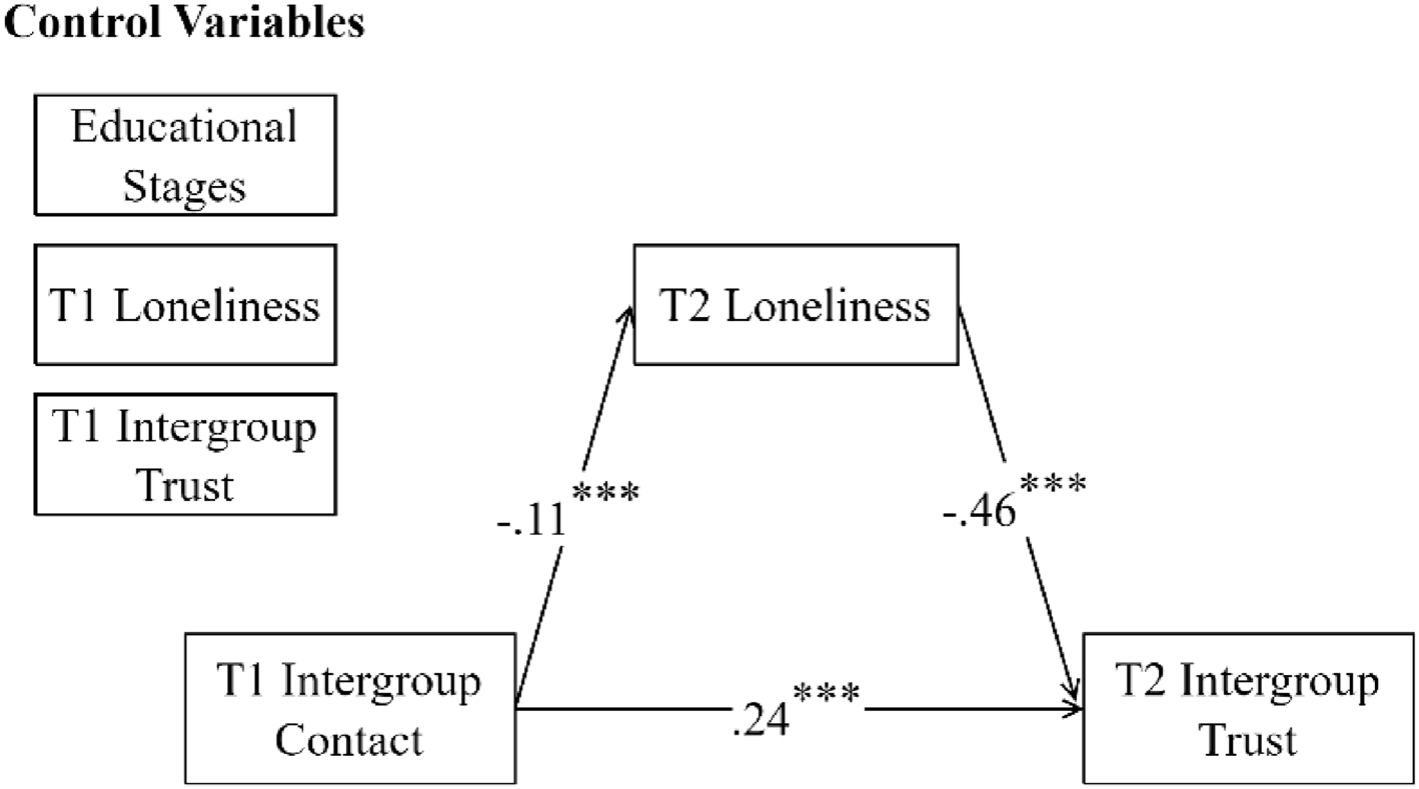
A longitudinal mediation model of intergroup contact, loneliness, and intergroup trust.

Building on the cross-lagged relationships between variables, the mediating effect of loneliness on the longitudinal association between intergroup contact and trust was further examined. A mediation model was constructed with T1 intergroup contact as the predictor, T2 intergroup trust as the outcome variable, T2 loneliness as the mediator, and educational stage, T1 loneliness, and T1 intergroup trust as control variables; see Fig. 2 for the model.

Results indicated that T1 intergroup contact could directly predict positive changes in intergroup trust, as well as indirectly influence T2 intergroup trust through its negative prediction of changes in loneliness. The mediating effect accounted for 18% of the total effect, with a 95% CI [0.020, 0.821].

## Discussion

### Development of intergroup contact and trust among adolescents in China’s ethnic regions

This study found that, within one year, intergroup contact among adolescents in ethnic regions had generally increased. Middle school students showed a significant increase in intergroup trust, while the level of intergroup trust among high school students remained unchanged, mostly supporting Hypothesis 1. The increase in adolescent intergroup contact may be the result of schools actively promoting and conducting interethnic interaction activities. These activities create opportunities for adolescents from different ethnic groups to understand each other, cooperate, and communicate, laying the foundation for the formation of cross-ethnic friendships. The divergent trajectories in intergroup trust across educational stages point to a developmental variation in the evolution of interethnic attitudes. The observed augmentation in trust among middle schoolers resonates with the findings of Vezzali et al.^10^ and Zalk et al.^11^, which underscore the positive trajectory of adolescent interethnic relations. Within the context of the macro sociocultural environment of China, augmented by the deliberate structuring of intergroup contact within school curricula, middle school students may benefit from enhanced opportunities for meaningful intergroup interactions, thereby bolstering their trust in outgroup members. Conversely, while high school students initially exhibit higher levels of intergroup trust relative to middle school students, their trust does not significantly shift over the observed period. This stability could be reflective of the ongoing maturation process, as it is generally acknowledged that adults exhibit more entrenched intergroup attitudes. This premise is further corroborated by the work of Mana^44^, which delineated that Jewish and Arab university students’ attitudes towards outgroups remained unaltered following a year of intergroup contact, illustrating the potential for attitudinal stabilization as individuals advance in age.

### The dynamic relationship between intergroup contact and trust

The cross-lagged analysis indicates that intergroup contact among adolescents in ethnic regions significantly predicts intergroup trust one year later, while the reverse is not observed, suggesting that intergroup contact is a precursor to intergroup trust. This finding is consistent with the research by Dhont et al.^25^ and supports Hypothesis 2 of the present study, providing new evidence for the contact hypothesis^15^ within the context of Chinese social and cultural backgrounds. Under the influence of the Chinese government’s vigorous policies promoting interethnic communication, interaction, and integration, as well as the shared overarching identity of the Chinese nationality among all ethnic groups, intergroup contact may have a stronger policy-driven foundation and basis for interaction compared to Western immigrant societies. This could also be more conducive to enhancing intergroup trust among adolescents in China’s ethnic regions. On the other hand, the selection bias effect was not supported in this study. This may be because the selection bias effect is more applicable to contexts with tense intergroup relations, where prejudice and hostility towards outgroups affect the willingness for intergroup contact. In the social and cultural context of China’s ethnic regions, adolescents generally possess a higher level of intergroup trust, with their opportunities for intergroup contact likely being more constrained by environmental opportunities than by subjective willingness to engage in interethnic interactions. Therefore, intergroup trust did not significantly predict intergroup contact experiences one year later.

### The mediating role of loneliness in the relationship between intergroup contact and trust

Cross-lagged analysis reveals that intergroup contact is a precursor variable to loneliness, and loneliness is a precursor variable to intergroup trust, providing support for the pathway in which intergroup contact affects intergroup trust through the mediator of loneliness. Further mediation effect testing, controlling for baseline loneliness and intergroup trust, indicates that intergroup contact can directly predict positive changes in intergroup trust, as well as indirectly influence changes in intergroup trust through its negative prediction of changes in loneliness, supporting Hypothesis 3 of this study. This suggests that within the socio-cultural context of China’s ethnic regions, intergroup contact not only possesses the unique quality of transcultural interaction but also has the positive function of interpersonal communication. In an environment where China is vigorously promoting cross-ethnic interactions, contact with peers from other ethnicities provides adolescents in ethnic regions with broader social interaction and support^32^. This may reduce feelings of loneliness through the establishment of cross-ethnic friendships and the formation of a shared identity^37^. This decrease in loneliness helps adolescents enhance interpersonal trust and generalize trust at the individual level towards friends from other ethnicities to positive attitudes towards the entire out-group^30^. It also alleviates fears and anxieties associated with intergroup interactions^29^, thereby enhancing trust in the out-group.

### Research implications

The findings of the study offer several implications for fostering the development of intergroup trust among adolescents in ethnic regions. First and foremost, it is essential to persist in promoting contact and interaction between different ethnic groups. The study provides substantial research support for the implementation of China’s mainline strategy to “promote communication, interaction, and integration” among all ethnicities. Even within the context of Chinese socio-cultural backgrounds, the positive changes in adolescent intergroup attitudes do not occur unconditionally or naturally; intergroup contact is a vital prerequisite for attitude improvement. Schools can promote intergroup contact through various means such as multicultural education, organizing cross-ethnic sports and cultural events, interethnic group cooperative learning both inside and outside the classroom, and mutual visitation and exchange programs. Secondly, the key period for the development of intergroup trust among adolescents in ethnic regions within the Chinese socio-cultural context may lie in the juvenile stage. Therefore, it is particularly necessary to strengthen the environment for cross-ethnic interactions during the middle school stage. It is crucial to integrate resources from families, schools, communities, and other sectors to collaboratively create positive opportunities for intergroup contact among adolescents of various ethnicities. Thirdly, given the significant bridging role of loneliness between intergroup contact and trust, it is imperative to focus on the depth of intergroup interactions and to initiate a diverse range of activities designed to foster cross-ethnic friendships among adolescents from different ethnicities during intergroup encounters. This would serve to alleviate the emotional aspect of loneliness, thereby reinforcing the positive influence of intergroup contact on intergroup trust.

### Limitations and future directions

This study has several limitations and areas for improvement. First, due to the limited number of follow-up measurements, the study has only revealed changes and relationships at the aggregate level, which may obscure individual differences. Future studies should continue with more time points and differentiate between inter-individual and intra-individual levels using random intercept cross-lagged panel models. Second, the research was restricted to two schools in ethnic regions, which makes it difficult to compare the effects of micro-environmental differences across schools. The study also failed to differentiate between intergroup contacts that adolescents engage in at school and those in external locations, such as communities, making it difficult to determine the unique contribution of intergroup contact in the school setting to intergroup trust. Future research should further specify the context of intergroup interactions. Third, due to sampling difficulties, this study was conducted only in two ethnic regions of Sichuan Province, China, primarily involving Tibetan, Qiang, Yi, and Han ethnic groups. Whether the research conclusions can be generalized to other ethnic regions and groups in China remains to be further investigated.

### Electronic supplementary material

Below is the link to the electronic supplementary material.

Supplementary Information 1.

## Data Availability

Data is provided within supplementary information files.

## Intergroup contact experience scale

**Table:**

Item	
1. The frequency of my contact with people from other ethnic groups in my academic life	1 never–7 frequently
2. The frequency of my contact with roommates from other ethnic groups	1 never–7 frequently
3. The frequency of my conversations with classmates from other ethnic groups	1 never–7 frequently
4. The frequency of my visits to the residences of classmates from other ethnic groups	1 never–7 frequently
5. The number of friends from other ethnicities among my friends	1 almost none–7 A large proportion
6. My relationship with people from other ethnic groups is distant/close	1 distant–7 close
7. My relationship with friends from other ethnic groups is competitive/cooperative	1 competitive–7 cooperative
8. My relationship with people from other ethnic groups is equal/unequal	1 unequal–7 equal
9. My relationship with friends from other ethnic groups is pleasant/unpleasant	1 unpleasant–7 pleasant

## Intergroup trust scale

**Table:**

Item	
1. Trust	1 never feel this way–7 always feel this way
2. Reliability	
3. Lack of trust	
4. Suspicion	

## UCLA loneliness scale

**Table:**

Item	
1. How often do you feel that you are “in tune” with the people around you?	1 never–4 always
2. How often do you feel that you lack companionship?	
3. How often do you feel that there is no one you can turn to?	
4. How often do you feel alone?	
5. How often do you feel part of a group of friends?	
6. How often do you feel that you have a lot in common with the people around you?	
7. How often do you feel that you are no longer close to anyone?	
8. How often do you feel that your interests and ideas are not shared by those around you?	
9. How often do you feel outgoing and friendly?	
10. How often do you feel close to people?	
11. How often do you feel left out?	
12. How often do you feel that your relationships with others are not meaningful?	
13. How often do you feel that no one really knows you well?	
14. How often do you feel isolated from others?	
15. How often do you feel you can find companionship when you want it?	
16. How often do you feel that there are people who really understand you?	
17. How often do you feel shy?	
18. How often do you feel that people are around you but not with you?	
19. How often do you feel that there are people you can talk to?	
20. How often do you feel that there are people you can turn to?	
